# Predictors of antepartum and postpartum suicidal ideation at a diverse urban obstetric setting

**DOI:** 10.1186/s12889-026-26830-6

**Published:** 2026-03-03

**Authors:** Lauren A. Kobylski, Jennifer Keller, Ellen W. Yeung, Huynh-Nhu Le

**Affiliations:** 1 Department of Psychological & Brain Sciences, The George Washington University, 2013 H St NW, Washington, DC 20006 USA; 2 Department of Obstetrics & Gynecology, The George Washington University School of Medicine and Health Sciences, 2300 I St NW, Washington, DC 20037 USA

**Keywords:** Suicidal ideation, Perinatal, Antepartum, Postpartum, Risk factors

## Abstract

**Background:**

Perinatal suicidality is a leading contributor to maternal mortality, and is associated with adverse outcomes for birthing individuals and children. Further research is needed to establish risk factors for perinatal suicidal ideation (SI), particularly among diverse populations. This study explored risk factors for perinatal SI within a diverse urban obstetric setting.

**Methods:**

Data were derived from the electronic medical records of 3,983 perinatal individuals (41.8% Black/African American, 34.6% utilizing public health insurance) who completed the Edinburgh Postnatal Depression Scale (EPDS). 2,297 antepartum screens and 1,686 postpartum screens were assessed for SI via a positive endorsement of the EPDS’ item 10. Bivariate analyses were used to compare characteristics among individuals with and without SI, and multivariable logistic regression was used to examine predictors of SI.

**Results:**

Prevalence of antepartum and postpartum SI was 4.7% and 3.1%, respectively. After adjustment for race, ethnicity, employment status, type of health insurance, and age, unemployment (*aOR* 3.01, 95% *CI* 1.06–8.50, *p*=.038) and lifetime mood disorder diagnosis (*aOR* 5.05, 95% *CI*: 1.89–13.48, *p*=.001) remained significant risk factors for antepartum SI. Prescription of psychiatric medications at screening (*aOR* 3.55, 95% *CI* 1.56–8.06, *p*=.003) was a significant predictor of postpartum SI after adjusting for type of health insurance, age, lifetime mood disorder diagnosis, term vs. preterm delivery, infant birthweight, and Neonatal Intensive Care Unit (NICU) admission.

**Conclusions:**

Findings suggest that systematic and comprehensive screening in obstetric practices will facilitate the identification of individuals at risk for SI. Further research is needed to develop preventive interventions to mitigate perinatal SI risk.

## Background

Suicide in the perinatal period (pregnancy to the first year postpartum) is a significant clinical and public health issue. Suicide and overdose are leading causes of death for individuals in the first year following childbirth [1, 2]. Globally, up to 20% of deaths in the first year postpartum are due to suicide [3]. Suicidal ideation (SI) is a well-established predictor of suicide attempts and deaths in the general population [4, 5]. In perinatal individuals, rates of SI are concerning; the pooled prevalence of perinatal SI is estimated at 8% (10% during pregnancy and 7% in the postpartum period) [6]. Moreover, rates of SI during pregnancy have risen over time, with one study citing an increase of 100% from 2008 to 2018 [7]. Racial disparities appear to be worsening, with Black birthing individuals experiencing the sharpest rise (a 700% increase) in prenatal SI [7]. Over a similar time period (2006 to 2017), another study reported that the largest proportional increases in perinatal suicidality were among Non-Hispanic Black individuals, individuals with lower incomes, and younger individuals [8].

Perinatal suicidality (e.g., suicidal thoughts, behaviors, attempts, and deaths) have been associated with a constellation of adverse outcomes for birthing individuals and their children [9–11]. Perinatal SI has also been associated with longer-term consequences, including poor mother-infant interactions [12, 13]. Given the tragic consequences associated with perinatal suicidality, prediction of risk is of utmost importance for the long-term well-being of parents, children, and families. With appropriate screening and intervention, suicide is preventable, making an increased focus on understanding the risk factors for perinatal SI necessary for reducing maternal mortality.

Existing research on perinatal SI has focused on establishing baseline prevalence rates and identifying demographic and psychological risk factors. Reported rates of SI during pregnancy have ranged widely [14], varying across clinical samples and recruitment settings. For instance, in the United States (U.S.), the prevalence of perinatal SI is over 20% in obstetric clinics serving a predominantly Black urban population [15] and in programs treating neuropsychiatric illness during the perinatal period [16]. These higher rates in select samples and settings are consistent with current knowledge of established risk factors for perinatal SI, which include younger age, being unmarried, exposure to violence, and personal or family history of psychiatric disorders [17, 18]. However, to date, few studies have examined the role of obstetric and neonatal complications as risk factors for SI in the perinatal period. Pregnancy complications, broadly defined, have been correlated with postpartum SI in one racially and ethnically diverse urban sample of 1,080 women [19]. A different study associated severe vaginal lacerations and planned Cesarean deliveries with increased postpartum SI risk among 8,394 individuals (the majority of whom were Caucasian, partnered, and using private health insurance) receiving obstetric care at a large suburban health system [20]. However, other obstetric- or neonatal-related risk factors have yet to be identified or confirmed, suggesting a need for additional research in this area.

The study of perinatal SI is complex for a number of reasons. Compared to perinatal mood and anxiety disorders, which are the most common complication of pregnancy and childbirth and affect up to one in five perinatal individuals [21, 22], perinatal SI is a less frequent phenomenon. Additionally, measurement of perinatal SI varies in terms of the construct studied (e.g., thoughts of self-harm, thoughts of suicide), number of screening items, and method of administration (e.g., self-report, clinician-assessed) [23]. Furthermore, existing literature ranges across types of samples, geographic locations, and diversity, and it is therefore unknown how generalizable findings are to a broader population. Heterogeneity in the study of perinatal SI precludes direct comparison of risk factors across studies. Finally, most research has focused on either SI during pregnancy or the postpartum period, with few studies examining both time periods, thus making comparison of differential risk difficult.

Overall, perinatal SI is vastly understudied, with a high prevalence and negative outcomes, and more information regarding risk factors is needed to better identify at-risk patients and those who may benefit most from prevention and treatment. Much of the existing literature has captured risk factors for perinatal SI, rather than differentiating SI in pregnancy versus the postpartum period. Such knowledge will be critical in informing the implementation of effective suicide prevention programs for perinatal individuals, which have yet to be developed and are urgently needed [24]. The purpose of the current study is to examine potential predictors of antepartum and postpartum SI across multiple domains. Additionally, the present analysis draws upon a diverse urban sample, which will contribute to researchers’ and practitioners’ ability to address widening disparities in rates of perinatal SI.

## Methods

### Sample and procedures

**Fig. 1:**
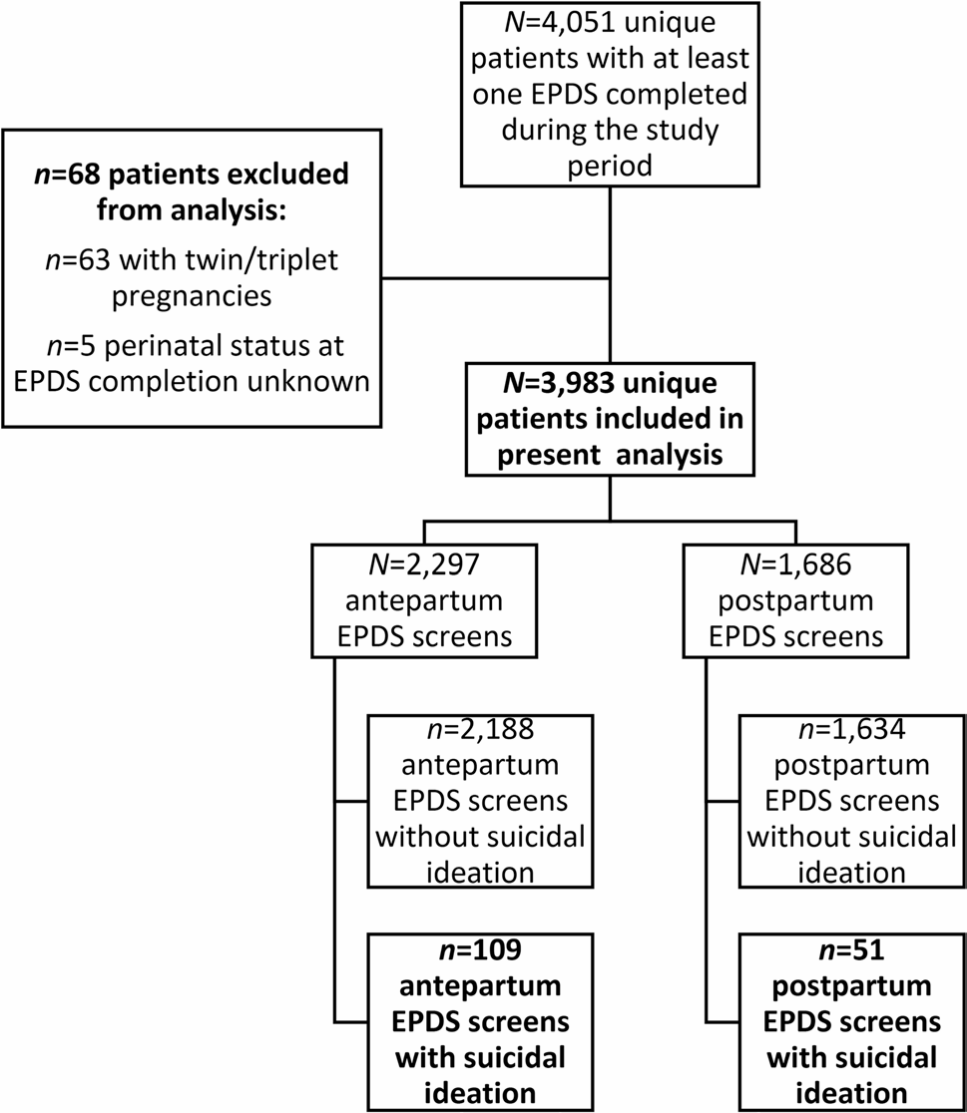
Selection of analytic sample

Data were derived from the electronic medical records (EMR) of an urban obstetric clinic in the U.S. with a universal depression screening protocol from the time of integration of depression screening into the EMR in the fall of 2021 to the start of the study in the fall of 2023. Criteria for inclusion included completion of the Edinburgh Postnatal Depression Scale (EPDS) [25] at least once during pregnancy or the postpartum period within the clinic. Individuals with non-singleton pregnancies were excluded due to the greater risk of complications, increased medical complexity, and closer monitoring compared to those with singleton pregnancies. An additional exclusion criterion included if it was unclear whether an EPDS was completed during the antepartum or postpartum period (i.e., there was no date associated with the EPDS). EPDS screens with any missing item responses were treated as missing data and thus excluded from analyses. Clinical protocol specified EPDS administration at the initial prenatal visit, the 28-week prenatal visit, and the routine 6-week postpartum visit, when applicable; most screens were self-administered via electronic or paper format and subsequently reviewed by the obstetric provider. In the case that multiple EPDS screens were available for a patient, the screen with the highest item 10 score (or, if no SI was endorsed, the highest total EPDS score) was selected for analysis. Selection of the analytic sample is depicted in Fig. 1.

All study procedures were approved by the George Washington University Institutional Review Board, which also waived consent to participate. Individuals who reported SI or clinically significant levels of depression were followed and referred to further assessment and psychological services based on level of suicide risk and clinical judgment.

### Measures

The primary outcome of interest was endorsement of SI during the antepartum or postpartum period, as measured by the EPDS. The EPDS is a widely used and validated instrument assessing perinatal depressive symptoms. [25] Item 10 on the EPDS specifically assesses SI in the preceding week with the following statement: “*The thought of harming myself as occurred to me*.” Respondents answer according to a four-point Likert scale with the following options: “*never*” (0), “*hardly ever*” (1), “*sometimes*,” (2), or “*yes*,* quite often*” (3). This item is commonly used within both obstetric practices and in perinatal mental health research to indicate the presence of SI. Participants who had a score of ≥1 on this item at least once during the perinatal period were considered to have endorsed SI, consistent with previous literature [26, 27, 20, 28–30].^1^

Potential predictors of SI endorsement were examined across multiple domains, including demographic (race, ethnicity, employment status, health insurance, and age), psychological (lifetime mood disorder diagnosis and prescription of psychiatric medications at screening), obstetric (prior pregnancy (regardless of outcome), history of abortion or miscarriage, term vs. preterm delivery, and delivery method), and neonatal (sex assigned at birth, birthweight, Apgar scores at one and five minutes of life, and Neonatal Intensive Care Unit (NICU) admission) characteristics. All demographic variables and lifetime mood disorder diagnosis were obtained from patients’ medical histories and problem lists, which predated EPDS screening. Prescription of psychiatric medication reflected active medications listed in patients’ records at the time of EPDS completion. All obstetric and neonatal variables were derived from clinical records associated with the perinatal period during which the EPDS was completed. Potential predictors related to delivery and neonatal outcomes were included only in postpartum EPDS analyses.

### Data analysis

Descriptive statistics were used to describe overall sample characteristics. To determine whether there were statistically significant differences in potential predictors between SI endorsers and non-endorsers, bivariate analyses (i.e., Chi-square tests, Fisher’s exact tests, *t* tests) were used. Multiple logistic regression was conducted to identify significant predictors of SI after controlling for all identified covariates. Inclusion of variables in the final model was based on statistically significant differences resulting from bivariate analyses. Statistical significance was defined as *p*<.05 for bivariate and multiple regression analyses. The preceding steps were completed for antepartum and postpartum SI separately, given the possibility that different risk factors may play differing roles in each time period.

## Results

The overall sample (*N* = 3,983) was racially diverse, consisting of 39.8% White/Caucasian individuals, 41.8% Black/African American individuals, and 18.4% identifying with other racial groups. The majority of the sample was employed (86.3%), though a sizable number were utilizing public health insurance (i.e., Medicaid, Medicare; 34.6%). The mean age of the sample was 33.1 (*SD* = 5.6). Approximately one-tenth of the sample had a lifetime mood disorder diagnosis (10.7%), and a similar proportion were prescribed psychiatric medications at screening (10.1%). The average total EPDS score of the entire sample was 6.1 (*SD* = 5.0), and 160 total individuals endorsed SI (4.0%).

### Antepartum suicidal ideation

The antepartum sample included 2,297 unique patients; 109 cases of SI endorsement (4.7%) were identified. Individuals with antepartum SI were significantly more likely to be Black/African American (*p*<.001), non-Hispanic (*p*=.013), unemployed (*p*=.001), and publicly insured (*p*<.001) compared to those with who did not endorse SI. Antepartum SI endorsers were significantly younger on average (*p*<.001) and more likely to have a lifetime mood disorder diagnosis (*p*<.001) and be prescribed a psychiatric medication at screening (*p*<.001) (Table 1).

**Table 1:** Characteristics of patients with antepartum EPDS screens

		Entire Sample (*N* = 2297)	Suicidal Ideation (*n* = 109)	No Suicidal Ideation (*n* = 2188)	*p*
Race, *n (%)*	White/Caucasian	749 (34.1)	17 (16.5)	718 (35.2)	**< .001**
	Black/African American	1122 (51.0)	77 (72.6)	1045 (49.9)	
	Other	328 (14.3)	12 (11.3)	316 (15.1)	
Ethnicity, *n (%)*	Non-Hispanic	1787 (93.7)	87 (100.0)	1700 (93.4)	**.013**
	Hispanic	121 (6.3)	0 (0.0)	121 (6.6)	
Employment, *n (%)*	Employed	495 (84.3)	13 (56.5)	482 (85.5)	**.001**
	Unemployed	92 (15.7)	10 (43.5)	82 (14.5)	
Health Insurance, *n (%)*	Private	1241 (55.7)	37 (34.6)	1204 (56.8)	**< .001**
	Public	986 (44.3)	70 (65.4)	916 (43.2)	
Age (years), *M (SD)*		32.1 (5.7)	30.2 (5.6)	32.2 (5.7)	**< .001**
Lifetime Mood Disorder Diagnosis, *n (%)*		291 (12.7)	39 (35.8)	252 (11.5)	**< .001**
Prescription of Psychiatric Medications, *n (%)*		222 (9.7)	29 (26.6)	193 (8.8)	**< .001**
Prior Pregnancy, *n (%)*		1748 (76.2)	81 (74.3)	1667 (76.3)	.642
History of Abortion/Miscarriage, *n (%)*		1093 (47.8)	55 (51.4)	1038 (47.6)	.444

The final multiple logistic regression model for antepartum SI included race, ethnicity, employment status, type of health insurance, age, lifetime mood disorder diagnosis, and prescription of psychiatric medications as predictors. After adjusting for all covariates in the model, unemployment (*aOR* 3.01, 95% *CI* 1.06–8.50, *p*=.038) and a lifetime mood disorder diagnosis (*aOR* 5.05, 95% *CI*: 1.89–13.48, *p*=.001) were significant risk factors for antepartum SI endorsement (Table 2).

**Table 2:** Multiple logistic regression results for antepartum SI

		Adjusted *OR*	95% *CI*	*p*
Race	Black/African American vs. White	0.94	0.15–5.97	.946
	Other vs. White*	0.00	NA	.997
Ethnicity	Hispanic vs. Non-Hispanic*	0.00	NA	.997
Employment Status	Unemployed vs. Employed	3.01	1.06–8.50	**.038**
Health Insurance	Public vs. Private	2.53	0.42–15.14	.310
Age		0.97	0.88–1.08	.600
Lifetime Mood Disorder Diagnosis		5.05	1.89–13.48	**.001**
Prescription of Psychiatric Medications		2.51	0.83–7.62	.105

*Results likely reflect sparse data, preventing reliable estimation of coefficients in the model. Consequently, these variables may not meaningfully contribute to the prediction of antepartum SI in this dataset

### Postpartum suicidal ideation

The postpartum sample consisted of 1,686 unique patients, 51 of whom (3.1%) endorsed SI. Individuals who endorsed postpartum SI were significantly more likely to be publicly insured (*p*=.022) and were younger on average (*p*<.001) than those who did not endorse SI. Postpartum SI endorsers were also more likely to have a lifetime mood disorder diagnosis (*p*=.009) and to be prescribed a psychiatric medication at screening (*p*<.001). Regarding obstetric and neonatal characteristics, individuals who endorsed postpartum SI were more likely to have had a preterm delivery (*p*=.002), lower birthweight infants on average (*p*=.006), and infants who were admitted to the NICU (*p*=.014) compared to those without SI (Table 3).

**Table 3:** Characteristics of patients with postpartum EPDS screens

		Entire Sample (*N* = 1686)	Suicidal Ideation (*n* = 51)	No Suicidal Ideation (*n* = 1634)	*p*
Race, *n (%)*	White/Caucasian	747 (47.9)	18 (37.5)	728 (48.2)	.332
	Black/African American	450 (28.9)	16 (33.3)	428 (28.7)	
	Other	362 (23.2)	14 (29.2)	348 (23.0)	
Ethnicity, *n (%)*	Non-Hispanic	1265 (89.4)	40 (88.9)	1224 (89.4)	.911
	Hispanic	150 (10.6)	5 (11.1)	145 (10.6)	
Employment, *n (%)*	Employed	465 (88.4)	17 (89.5)	448 (88.4)	1.000
	Unemployed	61 (11.6)	2 (10.5)	59 (11.6)	
Health Insurance, *n (%)*	Private	1287 (78.5)	32 (65.3)	1254 (78.9)	**.022**
	Public	352 (21.5)	17 (34.7)	335 (21.1)	
Age (years), *M (SD)*		34.3 (5.2)	31.8 (5.4)	34.4 (5.2)	**< .001**
Lifetime Mood Disorder Diagnosis, *n (%)*		134 (7.9)	9 (17.6)	125 (7.6)	**.009**
Prescription of Psychiatric Medications, *n (%)*		179 (10.6)	18 (35.3)	161 (9.9)	**< .001**
Prior Pregnancy, *n (%)*		1173 (69.6)	33 (64.7)	1139 (69.8)	.437
History of Abortion/Miscarriage, *n (%)*		687 (41.0)	21 (41.2)	666 (41.1)	.987
Gestational Age at Delivery, *n (%)*	Term	1474 (90.6)	39 (78.0)	1434 (91.0)	**.002**
	Preterm	153 (9.4)	11 (22.0)	142 (9.0)	
Delivery Method, *n (%)*	Vaginal	1012 (64.1)	33 (67.3)	978 (63.9)	.623
	C-section	568 (35.9)	16 (32.7)	552 (36.1)	
Infant Sex Assigned at Birth, *n (%)*	Male	821 (52.6)	25 (55.6)	795 (52.5)	.687
	Female	739 (47.4)	20 (44.4)	719 (47.5)	
Birthweight (grams), *M (SD)*		3256.4 (616.7)	2988.1 (708.8)	3264.4 (612.4)	**.006**
Apgar Scores, *M (SD)*	1 min	7.8 (1.4)	7.9 (1.0)	7.8 (1.4)	.678
	5 min	8.7 (1.0)	8.8 (0.4)	8.7 (1.0)	.604
NICU Admission, *n (%)*		171 (12.8)	11 (25.0)	160 (12.4)	**.014**

The final multiple logistic regression model for postpartum SI included type of health insurance, age, lifetime mood disorder diagnosis, prescription of psychiatric medications, term versus preterm delivery, infant birthweight, and NICU admission as predictors. Prescription of psychiatric medications (*aOR* 3.55, 95% *CI* 1.56–8.06, *p*=.003) was a significant risk factor for postpartum SI endorsement after accounting for the influence of other covariates in the model, and preterm delivery (*aOR* 3.46, 95% *CI* 0.95–12.67, *p*=.060) was marginally significant (Table 4).

**Table 4:** Multiple logistic regression results for postpartum SI

		Adjusted *OR*	95% *CI*	*p*
Health Insurance	Public vs. Private	1.00	0.37–2.96	0.997
Age		1.00	0.91–1.07	0.761
Lifetime Mood Disorder Diagnosis		1.01	0.29–3.60	0.983
Prescription of Psychiatric Medications		3.55	1.56–8.06	**0.003**
Gestational Age at Delivery	Preterm vs. Term	3.46	0.95–12.67	0.060
Birthweight		1.00	1.00–1.00	0.820
NICU Admission		1.40	0.56-3.51	0.473

## Discussion

The present study utilized EMR data from a diverse urban obstetric setting to identify predictors of SI endorsement. The rate of SI endorsement during pregnancy (4.7%) was higher than that of the postpartum period (3.1%). These prevalence rates are generally comparable to those identified in other U.S.-based reports [13, 20, 31–33]. Little research has explored potential explanations for observed higher rates of SI in the antepartum versus postpartum periods. It may be that the more frequent obstetric visits during pregnancy provide more opportunities for screening, and thus the lower observed rates of SI in the postpartum period are an artifact of fewer assessment timepoints or differing show rates for appointments in pregnancy versus postpartum. Additionally, pregnancy provides a longer-term period for assessment, whereas in the U.S., most patients have one routine visit around four to six weeks after delivery. It is possible that the prevalence of postpartum SI is elevated after this singular visit. Importantly, stigmatization of disclosing mental health difficulties and fear of involvement from child protective services in the postpartum period may also play a role. Prior research has examined individuals’ help-seeking behaviors for postpartum mental health difficulties, identifying salient concerns regarding adverse consequences of disclosure (e.g., being deemed an “unfit” parent, potential involvement of child welfare agencies [34–36]); such fears may also explain lower rates of postpartum SI. Future research should explore these hypotheses and elucidate contributors to and mechanisms of SI onset in these distinct time periods. Regardless, given that SI may lead to suicide attempts and deaths, identifying perinatal individuals experiencing or at risk for SI within the obstetric context is of critical importance. Our findings highlight the importance of repeated mental health screenings across the perinatal period; consistent administration and documentation of suicidality screens in obstetric care may represent an important step towards reducing maternal mortality due to suicide.

Our results suggest that distinct predictors exist for antepartum and postpartum SI risk. After adjustment, lifetime mood disorder diagnosis and unemployment significantly predicted antepartum SI, while prescription of psychiatric medications at screening was a significant predictor of postpartum SI. Notably, different psychological vulnerabilities were significantly associated with SI endorsement in the antepartum versus postpartum periods. A mood disorder history was significantly predictive of SI during pregnancy, perhaps due to heightened vulnerability resulting from hormonal changes or anticipatory stress, but prescription of psychiatric medications was not; this could be due to discontinuation or adjustment of antenatal medication use owing to concerns about fetal safety. Our finding that psychological risk factors significantly predict perinatal SI is generally consistent with existing literature [18]. Thus, given that current or past history of mental health difficulties is a risk factor for perinatal SI, routine mental health screenings during the perinatal period are essential for identifying childbearing individuals who may go on to develop SI. In clinical practice, endorsement of SI or the presence of significant depressive symptomatology should prompt a more comprehensive suicide risk assessment, safety planning if indicated, and facilitated referrals to mental health services.

We also identified unemployment as a relevant socioeconomic stressor predicting antepartum SI. Socioeconomic risk factors for perinatal SI have been previously examined using various proxies, including household income, type of health insurance, and education level [18]. To our knowledge, three studies have examined employment with respect to perinatal suicidality, finding no significant association of employment with postpartum SI in the United Kingdom [37], higher risk of antepartum SI or suicide attempt among those employed in South Africa [38], and more frequent unemployment among antepartum SI endorsers in Romania [39]. Given these discrepant findings, research should continue to explore how and why various aspects of socioeconomic status may contribute to perinatal SI risk with diverse samples. Additionally, clinicians and researchers may consider how connection to socioeconomic supports (e.g., employment resources, guidance accessing public health insurance benefits) during the perinatal period may reduce financial stress and mitigate suicide risk.

Our findings also indicate that individuals who deliver prior to 37 weeks gestation could potentially be at increased risk for postpartum SI. Prior research suggests that the experience of preterm labor and delivery, often an unexpected occurrence, is associated with psychological distress [40, 41]. Gestational age at delivery, however, did not significantly predict postpartum SI after adjustment in our results or in the findings of a different study [20]. In general, the literature on obstetric and neonatal risk factors for postpartum suicidality is mixed. For example, in one study, poor infant health in the first 45 days of life significantly predicted postpartum suicidal behavior [42]. However, in our results and others, proxies for infant health (e.g., birthweight, Apgar scores, NICU admission) were not identified as significant risk factors [20]. It is possible that obstetric and neonatal vulnerabilities are best represented by different risk factors across various samples and populations, and further research is needed to identify what type of screening for obstetric and neonatal complications in clinical practice will be most effective in identifying individuals at risk for postpartum SI.

Our study has several limitations. First, the cross-sectional design has implications for causal inference, and longitudinal studies in the future will be better positioned to determine whether the risk factors identified here cause antepartum or postpartum SI. Additionally, our sample was drawn from an urban obstetric clinic, potentially limiting generalizability to rural populations or to those who seek prenatal care and delivery outside of a traditional medical context. Similarly, our design limited access to only data retrievable from the EMR. Consequently, our closest proxies to psychological symptoms were limited, including lifetime mood disorder diagnosis and prescription of psychiatric medications at screening. Another limitation of our design is that we only had access to data for individuals who completed at least one EPDS screen; therefore, we do not know if or how suicide risk differs for non-screened individuals. Finally, we measured SI using a single item of the EPDS, so our results may underestimate true prevalence, as individuals with less frequent or severe SI may not have endorsed item 10. Of note, item 10 assesses frequencies of thoughts of harming oneself, which may not be interpreted as SI by all perinatal individuals [43]. Nevertheless, extensive research on perinatal SI uses this measure, as do many obstetric clinics across the U.S. and globally; our findings are thus comparable to a large majority of existing research on perinatal suicidality and implications are clinically relevant to the many healthcare settings implementing EPDS screening.

In conclusion, our results indicate that perinatal SI risk is multifaceted, and may shift across pregnancy and the postpartum period, suggesting the need for repeated screening and time-specific clinical responses based on these two distinct time periods. Routine mental health screenings in obstetric practice, and holistic assessment of an individual’s personal stressors (e.g., socioeconomic, obstetric) will aid in identifying at-risk individuals who will benefit most from referrals to mental health services and in preventing perinatal SI. The field of perinatal suicidality is relatively nascent, and future investigations into community- and societal-level risk factors and protective factors with longitudinal designs and diverse samples will significantly contribute to our ability to prevent perinatal suicide and improve the lives of perinatal individuals and their families.

## Data Availability

The dataset used and analyzed in the current study are available from the corresponding author on reasonable request.

## Abbreviations

EMR: Electronic medical record
EPDS: Edinburgh Postnatal Depression Scale
NICU: Neonatal Intensive Care Unit
SI: Suicidal ideation
US: United States

## Footnotes

1 Other researchers have used a ≥2 cutoff on this item to indicate SI endorsement [15, 31]; given the mixed state of extant literature, the broader cutoff was utilized to be more inclusive of potential perinatal SI.
